# Zwitterionic Photocurable Resin for High‐Resolution 3D Printing of Ultralow‐Fouling Microstructures

**DOI:** 10.1002/smtd.202501222

**Published:** 2025-11-04

**Authors:** Kun Wang, Natalie Hwee, Wade Degraff, Sophia M. Biener, Sijia Huang, Longsheng Feng, Seth Watts, William L. Smith, Bo Wang, Sourav Chatterjee, Tae Wook Heo, Jianchao Ye, Gang Cheng, Juergen Biener, Sangil Kim

**Affiliations:** ^1^ Department of Chemical Engineering University of Illinois at Chicago Chicago IL 60607 USA; ^2^ Lawrence Livermore National Laboratory Livermore CA 94550 USA; ^3^ Bishop O'Dowd High School Oakland CA 94605 USA

**Keywords:** 3D printing, metamaterial, projection stereolithography, ultralow‐fouling materials, zwitterionic photoresist

## Abstract

High‐resolution 3D printing technologies are enabling a new generation of microstructured materials for applications where biocompatibility is critical. However, most conventional 3D‐printable resins yield materials that exhibit trade‐offs between antifouling properties and mechanical robustness, limiting their applicability in living systems. In nature, zwitterionic surface groups form tightly bound hydration layers that act as effective barriers against protein and cell attachment. Inspired by this strategy, a zwitterionic acrylamide‐based photoresist—carboxybetaine di‐methacrylamide (CBDA)—is developed for projection‐based vat photopolymerization, enabling the fabrication of complex microarchitectures with exceptional antifouling properties. The bifunctional monomer allows the formation of dense, cross‐linked networks that resist swelling while maintaining a high density of zwitterionic groups. Printed structures exhibit strong resistance to protein and cell adhesion, as confirmed by porcine blood assays, alongside robust mechanical performance. As a demonstration, a tubular structure featuring a negative Poisson's ratio lattice is printed to showcase structural fidelity and versatility. This resin formulation offers a broadly applicable strategy for fabricating microscale devices and surfaces where antifouling performance and structural integrity are both essential—spanning biomedical interfaces, soft robotics, and beyond.

## Graphical abstract

A zwitterionic acrylamide‐based photoresist is developed for projection stereolithography, enabling the high‐resolution 3D printing of complex microstructures with intrinsic antifouling properties. This resin system combines mechanical robustness and zwitterionic surface chemistry without post‐functionalization, offering a versatile platform for fabricating bioinert microscale devices suitable for applications in soft robotics, diagnostics, and implantable technologies.

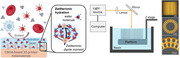

## 1 Introduction

In the past decade, three‐dimensional (3D) printing has garnered significant attention as a powerful platform for the cost‐efficient fabrication of complex structures with application‐specific geometries. Among various 3D printing technologies, projection stereolithography (PSLA) stands out for its ability to produce intricate macroscopic structures with micron‐scale resolution and high throughput.^[^ ^1^ ^]^ While the PSLA technology meets the throughput and resolution requirement for the cost‐efficient mass production of individualized, high‐precision parts,^[^ ^2^ ^]^ the development of photoresists that combine high‐resolution printability with strong surface‐resistance properties (e.g., antifouling properties) remains limited.^[^ ^3^ ^]^ This has created an urgent need for developing innovative photo‐chemistries that combine excellent printability and mechanical performance with the biocompatibility necessary for the fabrication of devices across a range of surface‐critical applications, including but not limited to biomedical devices.^[^ ^3^, ^4^ ^]^

Many 3D‐printed materials, particularly those interfacing with biological fluids or fouling‐prone environments, require surfaces that resist nonspecific protein and biomolecule adhesion. For such applications, the interfacial properties of the material are just as critical as its mechanical performance. Poly(ethylene glycol) (PEG) has long been considered as a candidate for providing non‐fouling surfaces,^[^ ^5^ ^]^ and PEG‐based resins have been incorporated into photocurable resin to improve biocompatibility.^[^ ^6^ ^]^ However, PEG‐based materials have shown limitations, including insufficient inhibition of protein adsorption^[^ ^7^, ^8^ ^]^ and oxidative instability, which can lead to degradation and foreign body responses.^[^ ^9^, ^10^ ^]^ These limitations of PEG‐based materials have motivated the search for alternative chemistries.

Zwitterionic materials, which mimic the design of phosphatidylcholine, the most common phospholipid in mammalian membranes, offer a promising solution.^[^ ^11^ ^]^ Their enhanced biocompatibility arises from overall charge neutrality and the strong hydration shell formed by zwitterionic groups, which together enable excellent resistance to biomolecular fouling.^[^ ^12^, ^13^, ^14^, ^15^ ^]^ Previous works have revealed zwitterionic materials with less than 5 ng/cm^2^ protein adsorption and prolonged blood compatibility.^[^ ^16^, ^17^, ^18^ ^]^ Several approaches have been developed to tether zwitterionic polymers on the surface of 3D‐printed biomedical devices through grafting.^[^ ^19^, ^20^ ^]^ While several studies have demonstrated that surface‐grafted zwitterionic polymers enhance resistance to fouling, these approaches often involve complex and time‐consuming surface modification steps, significantly hindering their widespread adoption for high‐volume production. To overcome the disadvantages associated with grafting methods, zwitterionic photocurable monomers have recently been developed that incorporate the zwitterionic functionality directly into 3D‐printed polymer networks.^[^ ^1^, ^21^, ^22^, ^23^, ^24^ ^]^ However, to date, only zwitterionic mono‐methacrylates have been synthesized, which are highly water‐soluble and unsuitable for aqueous environments.^[^ ^25^ ^]^ To address this, zwitterionic mono‐methacrylates are often blended with di(meth)acrylates or additional crosslinkers. Nevertheless, the resulting 3D‐printed zwitterionic structures retain hydrogel‐like characteristics, high water content and low mechanical stiffness, with a modulus typically below 3 MPa.^[^ ^21^, ^22^, ^24^, ^26^ ^]^ While acceptable for applications such as tissue engineering and biosensors, these properties are inadequate for high‐precision 3D printing of biomedical devices. Furthermore, the blending approach reduces the density of zwitterionic groups, thereby compromising antifouling efficacy and biocompatibility. To date, 3D‐printed zwitterionic structures have not demonstrated sustained resistance to thrombosis.^[^ ^21^, ^22^, ^23^, ^24^, ^26^ ^]^ Although one study reported the use of a zwitterionic dimethacrylate for 3D printing,^[^ ^18^ ^]^ the use of dimethacrylates in long‐term implantable medical devices remains limited due to their susceptibility to ester hydrolysis. A universal resin design for the fabrication of 3D‐printed biomedical devices that combines strong biocompatibility, good mechanical properties, and the potential for mass production has yet to be developed.

To address these limitations, we report the design, synthesis, and evaluation of a zwitterionic methacrylamide monomer, carboxybetaine di‐methacrylamide (CBDA), specifically engineered to form a stable, cross‐linked polymer network with a high density of zwitterionic groups. The strong electrostatic interaction between the carboxybetaine (CB) group and water leads to the formation of a rigid hydration shell that prevents surface‐induced conformational changes of the proteins and other biological foulants.^[^ ^27^ ^]^ The monomer was used to formulate a photocurable resin that provides excellent printability and biocompatibility. To demonstrate the potential of this CB‐based photocurable resin for printing intricate structures, a tubular metamaterial with negative Poisson's, resembling the functionality of a coronary stent, was 3D‐printed to highlight its suitability for microfabrication. 3D printed auxetic stents have recently attracted considerable interest due to the design flexibility enabled by additive manufacturing. For example, polyimide tests specimens based on a 2D re‐entrant chiral auxetic structure have been fabricated using a multi‐jet fusion (MJF) 3D printing process.^[^ ^28^ ^]^ Compared to the MJF 3D printing process, the PSLA process used in the present study offers a roughly 10× higher print resolution. Characterization of the PSLA 3D printed auxetic test structures fabricated with the CB‐based photocurable resin described in this work confirmed the expected negative Poisson's ratio behavior and demonstrated superior long‐term resistance to cell attachment compared to commercial metallic stents. This study establishes CBDA‐based photocurable resin as a versatile platform for applications requiring both mechanical robustness and surface antifouling, offering new design opportunities in advanced additive manufacturing beyond traditional uses.

## 2 Results and Discussion

**Figure 1:**
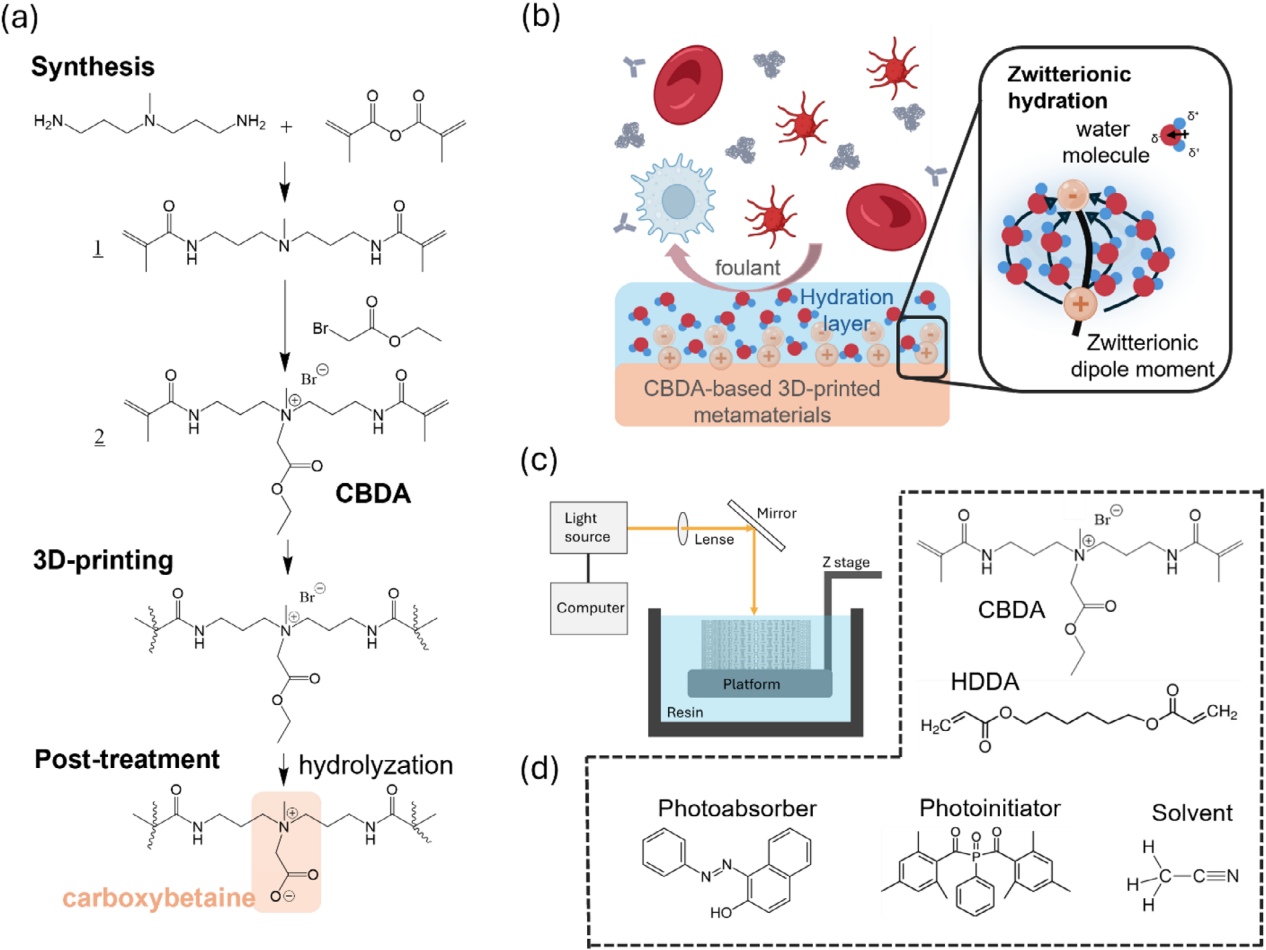
a) Process flow including CBDA synthesis, 3D‐printing, and post‐treatment used in this work. b) Illustration of the antifouling property of the 3D‐printed metamaterials from the CBDA‐based bioink. c) Schematic illustration of the PSLA technology that generates 3D structures by projecting a light pattern to induce localized polymerization within an ink reservoir. d) Components of the CBDA‐based photocurable resin.

The design of the CBDA monomer developed in this work was driven by the goal to optimize its anti‐biofouling and mechanical properties. The CBDA monomer was synthesized by aminolysis of methacrylic anhydride with 3,3’‐diamino‐N‐methyldipropylamine to form the methacrylamide functionality, followed by nucleophilic substitution of ethyl bromoacetate (**Figure** **1**a and experimental section). The chemical structure of CBDA was verified by ^1^H NMR spectroscopy (Figures S1a,b, Supporting Information). The zwitterionic CB functionality is generated after 3D printing by hydrolyzing the ester group of CBDA in an alkaline solution. This is made possible by using the acrylamide functionality, which overcomes the stability issues related to the hydrolysis of previous CB‐based methacrylate designs.^[^ ^29^ ^]^ The crosslinking functionality of the CBDA monomer allows for the formation of stiffer crosslinked polymer networks that resist the swelling typically observed in zwitterionic materials.^[^ ^16^ ^]^ To estimate the volumetric density of the zwitterionic CB groups, the Van der Waals volume (V_VdW_) of CBDA was calculated from its molecular formula (C_16_H_29_N_3_O_2_), applying the method described in ref. [30]. The calculated V_VdW_ of 325.38 Å^3^ translates into a volumetric density of 3.1 CBnm^−3^, from which an areal density of CB groups of >10^14^ cm^−2^ can be estimated on par with the areal density of zwitterionic groups in phosphatidylcholine (PC) lipid bilayers.^[^ ^31^ ^]^

CBDA contains a zwitterionic carboxybetaine group with a short spacer (consisting of a single ‐CH_2_‐ unit) between the negative and positive charges. This compact spacing is critical for achieving long‐term resistance to nonspecific protein adsorption, outperforming structures with longer carbon spacers.^[^ ^32^ ^]^ The short spacer length in CBDA supports the formation of a dense and stable hydration layer (Figure 1b), coupled with a relatively small electric dipole moment. Together, these features are expected to strongly suppress interaction with biofouling moieties.^[^ ^33^ ^]^

The CBDA monomer was integrated into a resin formulation for PSLA 3D printing (Figure 1c). The photocurable resin (Figure 1d and experimental section) contains 70% monomer in acetonitrile (ACN) along with a photoinitiator (Irgacure 819, 2 wt% of monomer) and a photoabsorber (Sudan I, 1.2 wt.% of the total monomer content) for optimal polymerization and print quality. ACN acts not only as a solvent for CBDA, which is a crystalline powder, but also facilitates the bulk hydrolysis of the ester functionality of the CBDA print to the active CB anti‐biofouling functionality by introducing nanoporosity (ranging from 2 to 5 nm, as confirmed by size exclusion test). Irgagure 819 was used as the photoinitiator to achieve high curing efficiency. We note that acyl‐phosphine oxide initiators may pose cytotoxicity concerns in bioprinting applications. In future work, we will explore alternative system—such as benzoxazine derivatives^[^ ^34^ ^]^ and ethyl (2,4,6‐trimethylbenzoyl) phenylphosphinate^[^ ^35^ ^]^ with improved cytocompatibility, and adjust initiator concentrations and post‐cure protocols.

**Figure 2:**
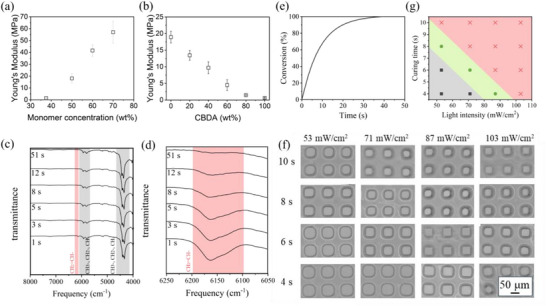
Characterization of the CBDA based photocurable resin: Young's modulus obtained from compression tests on poly(CBDA/HDDA) disk‐shaped samples as a function of a) the total monomer (CBDA+HDDA) content at a fixed CBDA to HDDA ratio of 4, and b) the CBDA/HDDA ratio at a fixed total monomer content of 37.5 wt.%. c) Near IR spectra of the CBDA‐based photocurable resin and d) detail of the vinyl overtone stretch region (6250–6050 cm^−1^) as a function of UV (405 nm) exposure time using a power density of 30 mW cm^−2^. e) degree of conversion versus UV exposure time calculated from the change in the near IR signal of the vinyl overtone stretch region. f) Optical microscope images and g) summary of a print resolution test using a pattern consisting of 50 µm holes and walls as a function of UV intensity and exposure time. Red, gray, and green regions in (g) represent over‐curing, insufficient curing, and good agreement with the print design.

Compression tests on bulk‐cured CBDA/HDDA‐based photocurable resin disks (**Figure** **2**a; Figure S2, Supporting Information) showed that increasing the ACN content (i.e., reducing the total monomer content) led to a decrease in Young's modulus and compressive strength. To mitigate this effect, we investigated the mechanical properties of CBDA/HDDA discs with varying CBDA/HDDA ratios while maintaining a fixed total monomer concentration of 37.5 wt.%. HDDA, serving as a short reactive crosslinker, is known for its minimal shrinkage, rapid polymerization rate, mechanical reinforcement, and chemical resistance.^[^ ^36^ ^]^ Compression tests confirmed that HDDA addition to the photocurable resin improves the mechanical properties of the 3D printed polymer network (Figure 2b). Additionally, biofouling tests (see below) indicated that a CBDA:HDDA ratio of ≥4 warrants a sufficiently high density of CB groups for high anti‐biofouling resilience.

The UV polymerization kinetics of the CBDA resin formulation described above were studied using near‐IR spectroscopy to monitor the temporal evolution of the vinyl group‐related absorption bands under UV irradiation (Figure 2c,d). Real‐time near‐IR spectra were collected from a drop of liquid photocurable resin placed on the detection window with a UV lamp positioned over the sample. Combination vibrations of CH_3_–, CH_2_–, and CH– were observed between 4200 and 4500 cm^−1^, while overtone vibrations of these three groups were found between 5600 and 6100 cm^−1^. The characteristic peaks related to the 2^nd^ overtone of the sp^2^‐CH stretch modes of the acrylamide vinyl group were observed ≈6170 cm^−1^.^[^ ^37^ ^]^ The polymerization kinetics were measured by monitoring the decrease in the intensity of the vinyl group‐related absorption bands as a function of UV exposure time. The main peak in the 4200 – 4500 cm^−1^ region, which corresponds to the CH_3_–, CH_2_–, and CH– absorption, remained unchanged during the photopolymerization reaction, thus providing an internal reference for quantifying the decrease of the C═C bond‐related peak area. Quantitative analysis of the vinyl group‐ related intensity (Figure 2e) revealed 69%, 91%, and 99% conversion of CBDA after UV exposures of 10, 20, and 40 s, respectively. The reaction kinetics is comparable to that of commercial SLA resins based on (meth)acrylate, (meth)acrylamide, or epoxy,^[^ ^38^, ^39^, ^40^ ^]^ demonstrating that CBDA photocurable resin polymerization kinetics is compatible with fast SLA‐based 3D printing.

To evaluate the printing performance of the CBDA resin in terms of lateral resolution, a test print pattern consisting of an array of 50 µm holes separated by 50 µm walls (Figure 2f) was designed and printed under a range of UV light intensities (53–103 mW cm^−2^) and exposure times (4–10 s). The results revealed that low light intensity and short exposure time led to insufficient curing of the projected patterns, while over‐curing of unprojected regions was observed at the highest light intensities and exposure times. For example, using a 53 mW cm^−2^ exposure for 4 s resulted in a wall thickness of only 35 µm instead of the designed 50 µm. On the opposite end of the spectrum, using 103 mW cm^−2^ exposure intensity for 10 s resulted in a wall thickness of 67 µm, and the designed square pattern became distorted due to over‐polymerization. The best printing performance in terms of resolution was achieved for short to medium UV exposure times (4–6 s) using UV light intensities in the 71–87 mW cm^−2^ range (Figure 2g). Overall, the best print results were obtained using a slightly higher UV dose (87 mW cm^−2^ for 8 s), as the increased UV dose promoted higher polymerization and improved the mechanical robustness and potential for applications, while still producing print dimensions close to the print design with minimal over‐polymerization. For printing conditions requiring lower UV doses or faster processing, a post‐curing strategy could be employed to further enhance polymerization and mechanical performance, which may be advantageous for applications demanding increased stiffness or durability.^[^ ^41^, ^42^ ^]^

**Figure 3:**
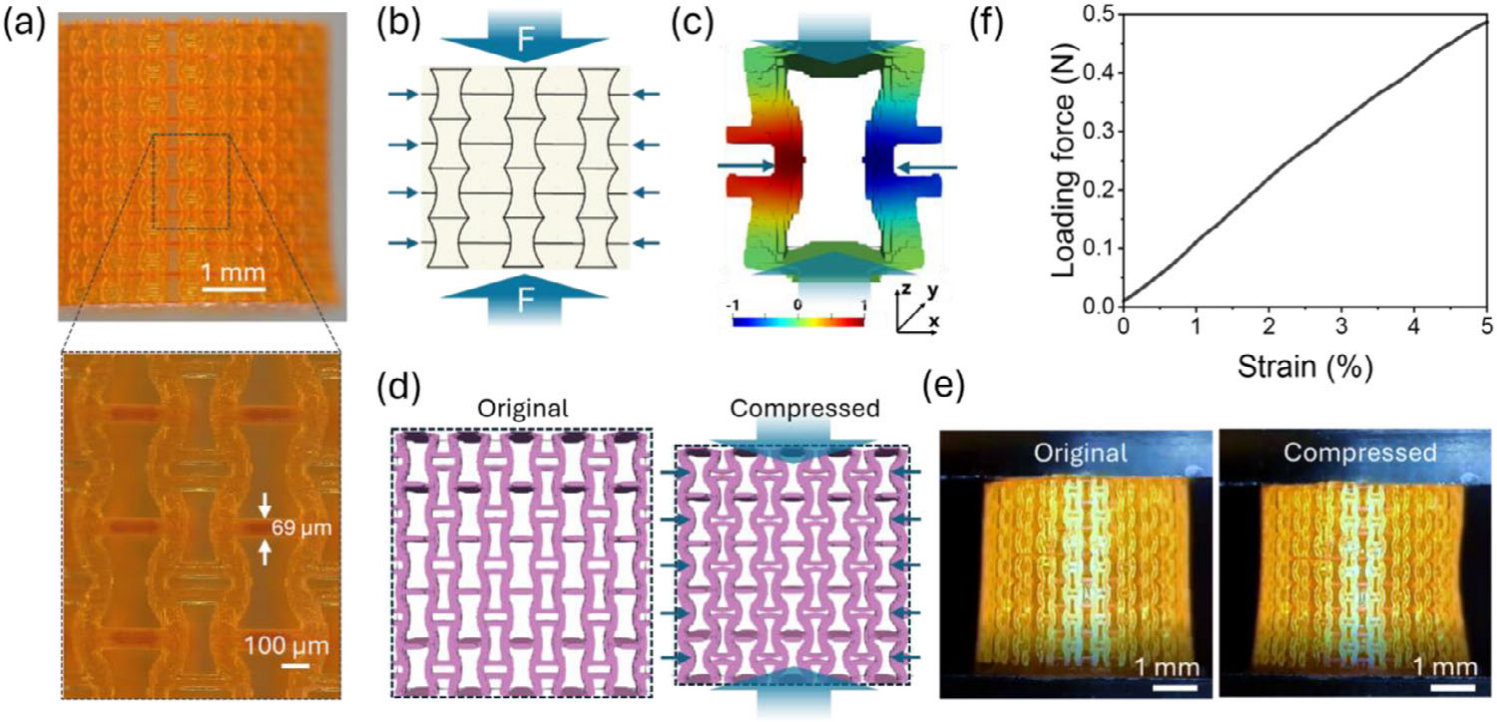
Negative Poisson's ratio stent structure design and test: a) Picture of the 3D‐printed stent. Inspection of the high‐mag image (bottom panel) reveals that both pores and overhangs are replicated with excellent precision with less than 10 µm overpolymerization in the vertical print direction; b) Negative Poisson's ratio re‐entrant honeycomb design. c) Numerical simulation showing the normalized displacement distribution along the x‐direction of one representative unit cell of the 3D printed meta‐structure, confirming the inward displacement of the center part of the unit cell under compressive stress of 0.2 MPa along z‐direction as expected for a negative Poisson's ratio behavior. d) Simulation of the compressive behavior of a  5 × 5 × 1 unit cell block yielding a Poisson's ratio of –0.92. e) Experimental compression tests on 3D printed structure and f) the corresponding stress‐strain curve. The Poisson's ratio obtained via image analysis from (e) is ‐0.93 in excellent agreement with the simulation.

Using the print parameters identified above, the vertical (*z*‐axis) resolution and potential overpolymerization of the CBDA photocurable resin were evaluated with a negative Poisson's ratio tubular design (**Figure** **3**a; Figure S3, Supporting Information). This structure incorporates densely packed narrow pores (≈50 µm) and overhanging beams, which are highly sensitive to local oxygen (inhibitor) depletion that can lead to overpolymerization. Comparison of the printed structures with the design dimensions showed excellent fidelity, with both pores and overhangs accurately replicated and overpolymerization limited to less than 10 µm in the vertical print direction.  Negative Poisson's ratio (auxetic) structures based on the re‐entrant honeycomb design (Figure 3b) are of interest in various engineering applications due to their unique deformation characteristics, such as enhanced energy absorption and indentation resistance. These properties make them attractive for diverse uses, including medical devices, soft robotics, flexible electronics, and impact‐mitigating materials.^[^ ^43^, ^44^, ^45^, ^46^ ^]^ In this study, to demonstrate the resin's capability to fabricate mechanically robust, intricate 3D structures, we selected a coronary stent‐inspired geometry featuring a negative Poisson's ratio. This metamaterial structure helps mitigate the risk of acute complications during the insertion of a coronary stent, as its outer diameter contracts when the structure is compressed along its tubular axis (Figure 3a). As described in detail in the experimental section, the design of the negative Poisson's ratio unit cell (Figure S4, Supporting Information) was developed by solving an inverse homogenization problem via topology optimization. For the developed 3D design, numerical simulations were conducted by applying a compressive stress of 0.2 MPa along the *z*‐axis of a single unit cell (Figure 3c). The simulation showed inward lateral displacement at the midpoints of the struts, confirming a negative Poisson's ratio behavior. An additional simulation on a larger structure composed of 5  ×  5  ×  1 unit cells (Figure 3d) yielded a Poisson's ratio of –0.92. These values closely match the experimental value of –0.93 determined from compression tests (Figure 3e,f). Optical images (Figure 3e) of the tubular negative Poisson's ratio test structure during compression to 5% strain confirmed the contraction of the diameter. The test structure exhibited a linear force‐strain curve up to 5% strain (Figure 3f), indicating good elasticity and impact resilience. The measured Poisson's ratio, calculated from the inward movement of the center part of the stent test structure, was ‐0.93 under an applied contact pressure of 0.045 MPa (Figure 3f), which agrees well with the simulation (‐0.92) and is on par with the Poisson's ratio reported from 3D printed polyimide auxetic stent test structures.^[^ ^28^ ^]^ Negative Poisson's ratio designs can provide stents with improved mechanical properties, reduced residual stresses, and less brittleness.^[^ ^47^, ^48^ ^]^ However, traditional stent fabrication based on laser cutting is limited to two‐dimensional designs, which restricts the design options for stents with negative Poisson's ratio.^[^ ^49^ ^]^ In comparison, our CBDA resin enables the creation of more complex 3D designs with excellent biocompatibility, without requiring complicated post‐treatment steps. These intricate designs and functionalities are impossible to fabricate using traditional manufacturing methods or other 3D printing technologies and require higher 3D printing resolution than that provided by MJF 3D printing process. The successful printing of the biomimetic, auxetic stent illustrates that the CBDA‐based resin could serve as a versatile platform for fabricating complex, biocompatible architectures with potential applications in implanted medical devices, hydrogel muscles, skin patches, and biosensors.^[^ ^50^, ^51^, ^52^ ^]^

**Figure 4:**
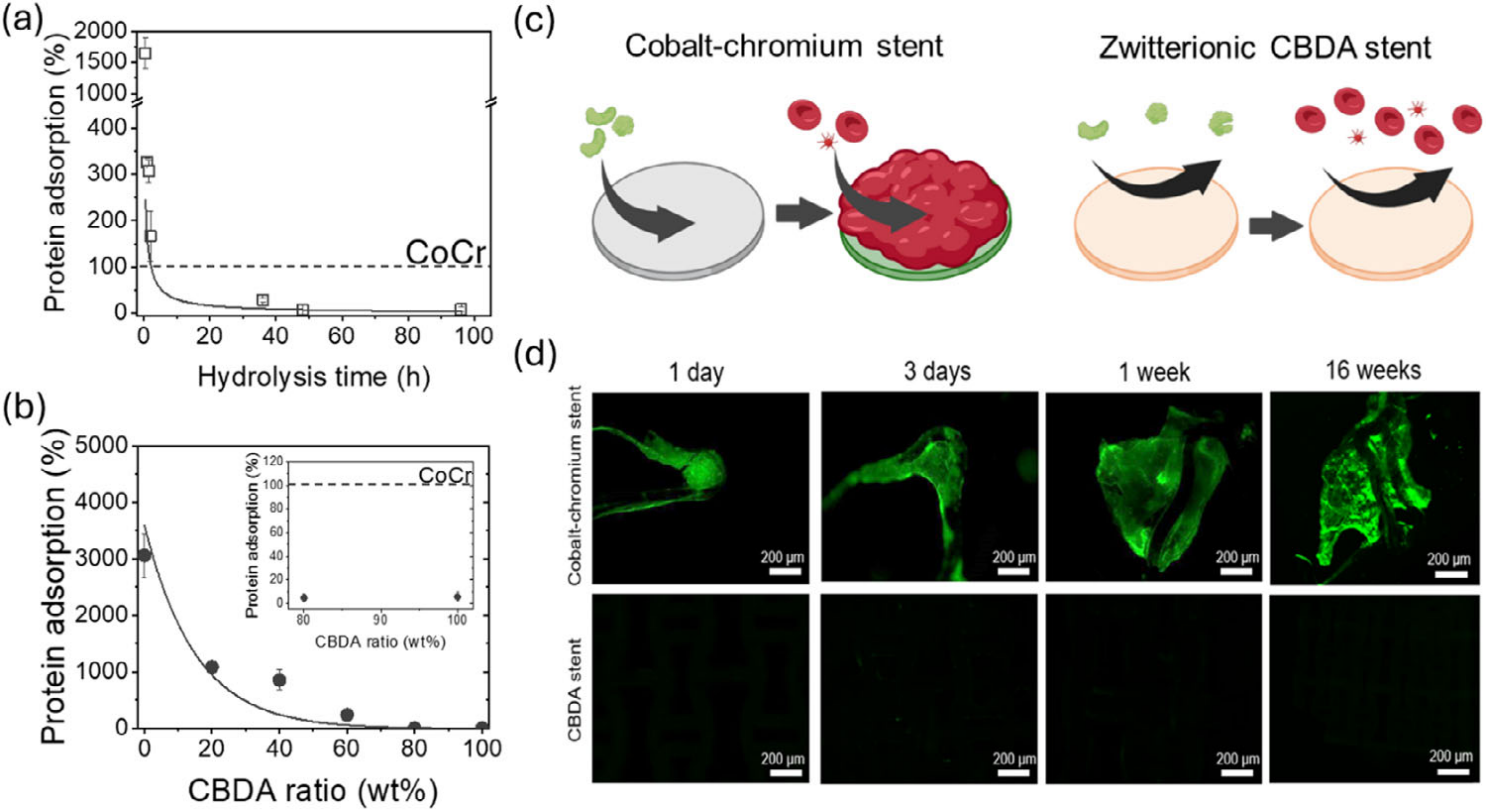
a) Surface protein adsorption of the pure CBDA film with different hydrolysis time. b) Surface protein adsorption of the cured CBDA‐based resin formulations with different CBDA/HDDA ratios after 96 h of hydrolysis. c) Illustration of commercial metal alloy stent and CBDA‐based 3D‐printed stent upon surface blood contact. d) Surface blood cell attachment on commercial metal alloy stent and CBDA‐based 3D‐printed stent.

Protein adsorption tests on the CBDA‐based negative Poisson's ratio tubular stent structure confirmed that the CB functionality effectively suppressed fibrinogen adsorption by more than 90% compared to a commercial CoCr coronary stent, which served as a control sample. Protein adsorption further enables other macromolecules within the blood to adhere to the surface. In the case of coronary stents, proteins rapidly adsorb onto the surface upon the initial blood contact, followed by the adhesion of blood cells, fibrinolysis, and coagulation. Fibrinogen (Fg) is widely used as a standard in vitro screening tool to assess protein adsorption levels, as it can strongly absorb onto various surfaces.^[^ ^53^, ^54^ ^]^ In this work, a fluorescence method was used to quantify Fg adsorption on the 3D‐printed CBDA stent structure sample. The adsorption of FITC‐labeled Fg on all samples was normalized to that of the CoCr control sample. Without the hydrolysis step required to generate the CB functionality, the CBDA test structure possessed a positively charged surface due to the quaternary amine groups and exhibited 1645 % higher Fg adsorption compared to the CoCr stent sample (**Figure** **4**a,b). Since proteins often possess distinct positively or negatively charged surface regions, charged material surfaces tend to increase the adsorption of the protein to the surface.^[^ ^55^ ^]^ As the CBDA hydrolysis time increased, the positively charged surface transitioned to a zwitterionic surface, drastically suppressing the adsorption of Fg on the CBDA sample surface. After 48 h of hydrolysis of the acetate groups, Fg adsorption on the CBDA sample surface was less than 8% of that on the CoCr stent, demonstrating the effective resistance of the CBDA to nonspecific protein adsorption. Increasing the hydrolysis time to 96 h did not further reduce protein adsorption.

While mixing CBDA with HDDA can improve the mechanical properties of poly(CBDA/HDDA) samples, it also reduces the density of zwitterionic CB groups. The effect of the CBDA/HDDA mixing ratio on surface protein adsorption was studied in a separate set of experiments (Figure 4b). Surfaces of negative Poisson's ratio tubular stent structures printed with 100% HDDA showed 30 times more protein adsorption compared to the CoCr stent. Increasing the CBDA concentration to 80 wt.% reduced protein adsorption to 18%, while pure CBDA sample decreased the protein absorption to 8%. This trend underscores the critical role of the density of zwitterionic CB groups in providing protection against protein adsorption through the formation of a tightly bonded hydration layer, which serves as a highly efficient barrier against protein absorption. This mechanism underpins the observed reduction in protein adsorption and the subsequent prevention of blood cell attachment (Figure 4c).

Cell adhesion experiments using porcine whole blood were conducted to further evaluate the resistance of CBDA‐based 3D printed structures to blood cell attachment. As shown in Figure 4d, blood cells began to agglomerate on the CoCr stent sample from the first day of blood contact. Several studies have also reported rapid cell attachment on CoCr stents within a short blood contact time.^[^ ^56^, ^57^ ^]^ In comparison, no attached cells were observed on the surfaces of CBDA‐based negative Poisson's ratio tubular structure sample after 16 weeks. These test results demonstrate that, even in a complex protein‐rich blood medium, cell attachments can be effectively prevented by the zwitterionic CB moieties in the CBDA‐based 3D‐printing materials.

## 3 Discussion and Conclusions

Our results illustrate that crosslinking zwitterionic monomers can facilitate the cost‐efficient, high‐resolution fabrication of complex microstructured components with excellent antifouling properties. Experiments with CBDA‐HDDA resin mixtures demonstrated a clear trend: anti‐biofouling properties improved with increasing zwitterionic group density, as evidenced by decreased protein adsorption and reduced blood cell adhesion. The printed structures replicated even the smallest design features with high fidelity and retained mechanical robustness across different CBDA concentrations, validating the suitability of the resin for projection‐based vat photopolymerization. Importantly, anti‐biofouling functionality was achieved without requiring post‐fabrication grafting or surface modification, except for a simple hydrolysis step to activate the CB zwitterionic groups. Furthermore, the ability to reliably fabricate architecturally complex structures—such as negative Poisson's ratio stent‐like lattices—demonstrates the geometric versatility and potential application breadth of the developed resin formulation.

While this study centers on materials development, the findings establish a foundation for future application‐driven optimization. Anti‐biofouling properties may be enhanced by increasing zwitterionic group density, such as by replacing 3,3’‐diamino‐N‐methyldipropylamine with 2,2’‐diamino‐N‐methyldiethyl to shorten the monomer backbone. Reducing the spacing between the charged groups could also improve hydration shell stability; for example, trimethylamine‐N‐oxide (TMAO) provides an even more stable hydration shell, resulting in better antifouling properties compared to the CB group used in the current design.^[^ ^33^ ^]^ For load‐bearing applications, higher modulus materials may be achieved through solvent‐free formulations or core‐shell architectures. Emerging multiwavelength stereolithography may support such designs by enabling fabrication of dense cores surrounded by antifouling shells, thereby preserving both mechanical integrity and surface functionality.^[^ ^58^ ^]^

In summary, this study presents a zwitterionic resin platform that enables high‐resolution additive manufacturing of ultralow‐fouling microstructures. The material design strategically balances printability, mechanical robustness, and intrinsic surface functionality, enabling broad applicability in technologies where antifouling performance is critical. Potential use cases include, but are not limited to, implantable medical devices, artificial muscles, soft robotics, and biosensors.

## 4 Experimental Section

### Materials

1,6‐Hexanediol diacrylate (HDDA), 4‐hydroxyanisole, methacrylic anhydride, Sudan I, phenylbis(2,4,6‐trimethylbenzoyl)phosphine oxide (Irg 819), and phosphate‐buffered saline (PBS), were obtained from Sigma‐Aldrich (Missouri, USA). 3,3’‐Diamino‐N‐methyldipropylamine was obtained from TCI (Tokyo, Japan). Ethyl bromoacetate and sodium hydroxide (NaOH) pellets were obtained from Thermo Fisher Scientific (Massachusetts, USA). Acetonitrile (ACN) was obtained from Beantown Chemical (New Hampshire, USA). Fluorescein isothiocyanate (FITC) isomer and human fibrinogen were purchased from Alfa Aesar (Haverhill, Massachusetts, USA). Ethylenediaminetetraacetic acid (EDTA) anticoagulant‐treated whole porcine blood was purchased from Sierra for Medical Science (California, USA). WGA stain was obtained from Biotium (Fremont, California, USA).

### Synthesis of Carboxybetaine‐Dimethacrylamide (CBDA)

DI water (150 mL) and ethanol (350 mL) were mixed in a 3 L three‐neck round bottom flask. NaOH (90.0 g, 2.25 mol) was added to the flask, and the system was cooled to 0 °C using an ice bath. 3,3’‐Diamino‐N‐methyldipropylamine (72.65 g, 0.5 mol) was introduced into the system, and 4‐hydroxyanisole (0.935 g, 0.0075 mol) was added as an inhibitor. Methacrylic anhydride (231.25 g, 1.5 mol) was pre‐dissolved in 500 mL ethanol and added to the system dropwise under nitrogen protection. The mixture reacted under stirring for 2 h at temperatures between 1°C and 4 °C, followed by 18 h reaction at room temperature. After the reaction, additional DI water (250 ml) and 4‐hydroxyanisole (0.935 g, 0.0075 mol) were added. A rotary evaporator was used to remove ethanol from the mixture, and the product was extracted using diethyl ether. After extraction, anhydrous sodium carbonate (10 g/200 ml of ether used to extract the product) was added to remove residual water. The ether solution was then filtered and additional 4‐hydroxyanisole (0.468 g, 0.0038 mol) was added. The diethyl ether was removed by a rotary evaporator to get the intermediate product (*1*) (Figure 1a). The intermediate product (*1*) (50 g) was then dissolved in acetonitrile to produce a 10 wt.% solution. Ethyl bromoacetate (1.2 mol/mol intermediate product) was added under nitrogen protection, and the system was allowed to react at 60 °C with stirring overnight. The resulting CBDA (*2*) was precipitated in diethyl ether, redissolved in water, purified by extraction with diethyl ether, and collected by freeze‐drying.

### Characterization of CBDA Polymerization Kinetics

The photopolymerization kinetics of the CBDA‐based photocurable resin were studied using in‐situ FT‐IR spectroscopy (Bruker Vertex 80, Bruker, Billerica, USA) equipped with an integrated 405 nm UV light source. FTIR spectra were recorded in the transmittance range of 4000–7000 cm^−1^, with a spectral resolution of 4 cm^−1^, 32 scans per spectrum, and a collection rate of 4 spectra per minute. Measurement of ambient air was set beforehand as a reference, and the sample curves were normalized with the peak of air moisture.

### Mechanical Testing of Bulk Poly(CBDA/HDDA) Samples

An EZ‐Test Compact Bench Testing Machine (Shimadzu, Japan) was used to test the mechanical properties of the poly(CBDA/HDDA) materials under compression. The samples were prepared by bulk UV curing (80 mW cm^−2^ for 15 min using a 405 nm source) and cut into disks with a diameter of 8 mm and a thickness of 2 mm. The disks were then compressed to failure at a compression rate of 0.5 mm min^−1^ using a 5000 N maximum load cell. The Young's modulus was obtained from the linear portion of the compression stress–strain curves. Compression tests of tubular negative Poisson's ratio test structures were also performed on the EZ‐Test Compact Bench Testing Machine (Shimadzu, Japan) using a compression rate of 1 mm min^−1^. Both poly(CBDA/HDDA) discs and stent structures were hydrolyzed for 96 h, equilibrated in water for at least one day before the mechanical testing, and tested in the hydrated state.

### Design of Negative Poisson's Ratio Structure

The negative Poisson's ratio tubular design was obtained by solving an inverse homogenization problem via topology optimization. In this approach,^[^ ^59^ ^]^ asymptotic homogenization theory is first applied to an initial design for the unit cell to compute the effective elasticity tensor of a material composed of a lattice of such unit cells. As noted elsewhere,^[^ ^60^ ^]^ once the derivations are complete, the actual computations require solving six “cell problems,” one per unique component of an initial strain tensor in three spatial dimensions, each subject to periodic boundary conditions, and then post‐processing these results to obtain the homogenized elasticity tensor. Next, the resulting elasticity tensor $C^{h}$ is used to form an objective function to be minimized. In this case, we computed the directional Poisson's ratios ν_*xz*_ and ν_*yz*_ by inverting the homogenzed elasticity tensor to obtain the homogenized compliance tensor $S^{h}$, applying a uniaxial stress in the *zz* direction, and dividing the *xx* and *yy* strains by the *zz* strain, then defined the objection function *f* as the sum of ν_*xz*_ and the square $\nu_{yz}^{2}$. This form of the objective function favors designs whose *xz* Poisson's ratio is as negative as possible, and whose *yz* Poisson's ratio is as close to zero as possible. Finally, in the last step, an adjoint sensitivity analysis is performed to compute the sensitivitites of the objective function *f* with respect to the design parameters that define the design (about which more below). For linear inverse homogenization problems, it can be shown that the adjoint solutions necessary to annihilate unknown derivatives in the sensitivity expression are the primal solutions to the cell problems,^[^ ^59^ ^]^ making this sensitivity analysis is very efficient. The objective function value and its sensitivities with respect to the design variables are then passed to a nonlinear programming optimization library and are iteratively reduced. In this work, we used Ipopt.^[^ ^61^ ^]^

The unit cell was discretized into 48 × 48 × 48  ≈ 110*k* hexahedral finite elements. In the classic “density parameterization” formulation^[^ ^62^ ^]^ of the topology optimization problem, the design was parameterized by assigning a “design density” ρ_*i*_ to each of the 110k finite elements. This design density is non‐dimensional and ranges in value between 0 and 1; when ρ_*i*_ = 1, finite element *i* has the material properties of a generic polymer base material (E = 1 GPa, ν = 0.4), and when ρ_*i*_ = 0, element *i* has much lower stiffness (E = 1 kPa) to simulate void. For intermediate values of ρ_*i*_, the material properties of element *i* are interpolated nonlinearly between these two extremes using the SIMP method,^[^ ^63^ ^]^ which tends to bias the design toward fully‐segregated phases. To enforce mechanical orthotropy on the homogenized elasticity tensor $C^{h}$, we enforced geometric orthotropy on its design, such that the design density ρ(*x*, *y*, *z*) = ρ(− *x*, *y*, *z*) = ρ(*x*, −*y*, *z*) = ρ(*x*, *y*, −*z*) within the unit cell; this is a sufficient but not necessary condition. This enforcement reduces the size of the design space by a factor of eight, such that there were 13,824 unique design variables. We note that it is possible to use symmetry methods to reduce the overall computational domain in like fashion,^[^ ^64^ ^]^ but in our implementation we used the full domain. We applied a standard Helmholtz filter ^[^ ^65^ ^]^ to impose a minimum length scale on the design space to ensure the problem was well‐posed. Since gradient‐based design algorithms can guarantee only local optimality, we launched dozens of design calculations from randomly‐selected starting points in the design space, and have reported the best result here. The final design was thresholded to force every element to have $\rho_{i}\in \left\{0,1\right\}$, and the phase boundary was extracted and converted into an STL file for further processing for the stent fabrication.

### Simulation of the Mechanical Response of the Negative Poisson's Ratio Structure

The mechanical response to the negative Poisson's ratio design was tested by numerical simulations, applying a compressive stress of 0.2 MPa to a 5 × 5 × 1 unit cell test structure along the z‐direction with the other two directions stress‐free. The mechanical equilibrium equation (∇ · σ_ij_ = ∇ · (C_ijkl_ε_kl_) = 0) was numerically solved using the Fourier‐spectral iterative‐perturbation scheme^[^ ^66^, ^67^ ^]^ to acquire the total displacement distribution within the solid ligaments at mechanical equilibrium under compression. The detailed numerical implementation is documented in refs. [66, 68]. The Young's modulus of the solid is assumed to be 40 MPa and Poisson's ratio as 0.35. For the pore region, all components in the elastic stiffness tensor are zero. The simulation system size is 320 × 320 × 64. The post‐processing of the optimized unit cell design used in this study is illustrated in Figure S4 (Supporting Information).

### 3D‐Printing of Zwitterionic Polymeric Stent

A Boston MicroFabrication S130 (Maynard, MA) printer was used to print the stent test structures with a 405 nm light source. The design was prepared as an STL file and then sliced with 10 µm layer thickness. The samples were printed on an indium tin oxide (ITO) coated glass slide to chemically detach the sample from the substrate to avoid mechanical damage. In most test prints, we used a resin formulation based on a blend of 56 wt.% CBDA, 14 wt.% HDDA, and 30 wt.% acetonitrile. Additionally, Sudan I was added at a concentration of 1.2 wt.% of the total monomer content and Irg 819 at a concentration of 2 wt.% of the total monomer content. Irgacure 819 was selected based on its absorption spectrum matching the emission spectrum of the PSLA printer used. The print parameters were optimized using a 50 µm × 50 µm test grid structure with varying UV light intensities and exposure times. The negative Poisson's ratio stent structure was printed with dimensions of 5 mm in diameter, 5 mm in length, and a wall thickness of 150 µm. After printing, the printed samples were cleaned in acetonitrile to remove uncured resin and then dried using a critical point dryer (Tousimis; Rockville, MD) prior to testing.

### Biocompatibility Test of the 3D‐Printed Zwitterionic Polymeric Stent

A fluorescence method was used to evaluate the protein adsorption on the pure CBDA film and cured CBDA‐based resin containing HDDA. In brief, the sample was equilibrated in PBS buffer for 20 min and then transferred to a 24‐well flat‐bottomed polystyrene plate. Each sample was treated with 1 mL of 1 mg mL^−1^ FITC‐labeled human fibrinogen (FITC‐Fg) solution. The samples were equilibrated in the FITC‐Fg solution for 30 min to allow protein adsorption. Subsequently, the samples were removed using a sterile tweezer and gently rinsed in PBS buffer three times to remove excess dye solution. The samples were then transferred to a glass slide and analyzed using an Olympus IX81 fluorescent microscope (Olympus, Japan) with a 20× objective lens through an FITC filter. Three samples were measured for each material under each hydrolysis condition. The exposure time was fixed at 200 ms for all samples. Ten images at different spots were taken for the surface of each sample. The fluorescence intensity of each image was analyzed by ImageJ software.

The blood compatibility of the 3D‐printed CBDA‐based stent was studied using undiluted porcine whole blood. The 3D‐printed stents were sterilized using an autoclave and then immersed in 100% fetal bovine serum (FBS) for 2 days. The treatment of FBS allows the preabsorption of serum proteins onto the surface, which promotes cell attachment and creates a situation that is closer to the in vivo experiments.^[^ ^69^ ^]^ Afterward, the stents were transferred to undiluted porcine whole blood. After each target test time period (e.g., 1 day, 1 week, 2 weeks), the stents were removed from the blood and gently rinsed in PBS buffer to eliminate loosely attached cells. Subsequently, the stents were stained by CF488A WGA and examined under an Olympus IX81 309 microscope (Olympus, Japan). For both the protein adsorption test and blood compatibility test, a commercial CoCr alloy stent (MULTI‐LINK VISION RX, Abbott, IL, USA) was used as a reference.

## Conflict of Interest

The authors declare no conflict of interest.

## Supporting information



Supporting Information

## Data Availability

The data that support the findings of this study are available from the corresponding author upon reasonable request.
