# Supplementary material for: Zwitterionic Photocurable Resin for High‐Resolution 3D Printing of Ultralow‐Fouling Microstructures
Source: Small Methods. 2025 Nov 4;9(12):e01222. doi: 10.1002/smtd.202501222 (PMC12716207; doi:10.1002/smtd.202501222)
Supplement: Supplementary file 1 — Supporting Information [file SMTD-9-e01222-s001.docx]

**Supplementary information**

**Zwitterionic Photocurable Resin for High-Resolution 3D Printing of Ultralow-Fouling Microstructures**

*Kun Wang, Natalie Hwee, Wade Degraff, Sophia M. Biener, Sijia Huang, Longsheng Feng, Seth Watts, William L. Smith, Bo Wang, Sourav Chatterjee, Tae Wook Heo, Jianchao Ye, Gang Cheng, Juergen Biener*, and Sangil Kim**

K. Wang, W. Degraff, S. Biener, G. Cheng, Sangil Kim

Department of Chemical Engineering, University of Illinois at Chicago, Chicago, IL 60607, USA

skim@uic.edu

N. Hwee, S. Huang, L. Feng, S. Watts, W. Smith, B. Wang, S. Chatterjee, T. Wook Heo, J. Ye, J. Biener

Lawrence Livermore National Laboratory, Livermore, CA 94550, USA

biener2@llnl.gov

S. Biener

Bishop O’Dowd High School, Oakland, CA 94605, USA

**Content**:

**Figure S1**: 1H NMR spectrum of CBDA and CBDA intermediates.

**Figure S2:** Compression tests of bulk cured poly(CBDA/HDDA) disks

**Figure S3**: Optical images of the 3D printed tubular negative Poisson’s ratio test structure

**Figure S4**: Post-processing the optimized unit cell design used in the numerical simulations

**
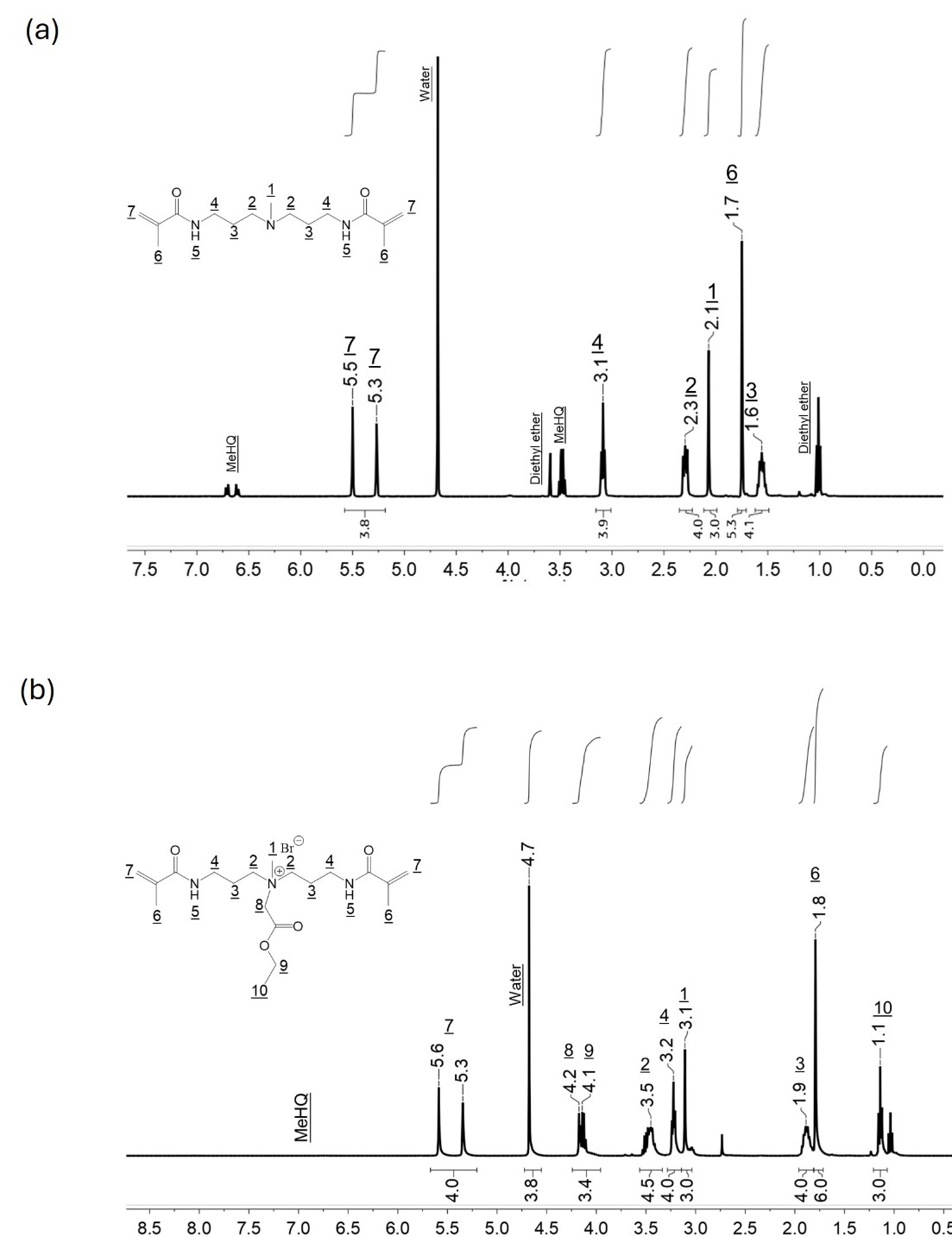
**

**Figure S1.** 1H NMR spectrum of a) intermediate product of CBDA and b) CBDA.

**
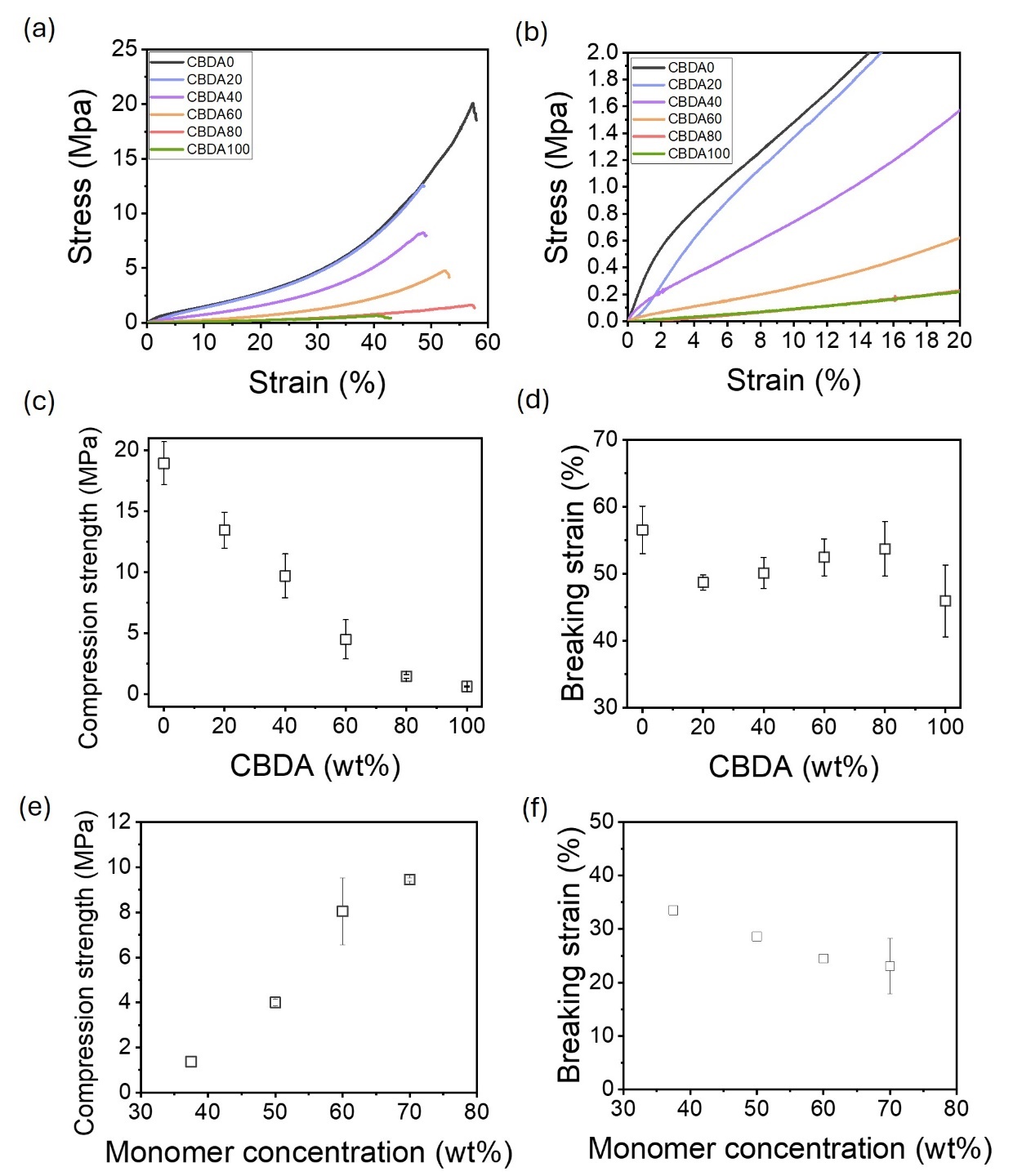
**

**Figure S2.** Compression tests of bulk cured poly(CBDA/HDDA) disks (8 mm diameter, 2 mm height): a) and b) Stress-strain curves and corresponding compressive c) and breaking strength d) data of poly(CBDA/HDDA) disks made from photoresist formulations with different CBDA/HDDA ratios using a fixed ACN content of 30 wt.%; e) compressive and f) breaking strength data of poly(CBDA/HDDA) disks prepared from photoresist formulations with different ACN contents but a fixed CBDA/HDDA ratio of 4 wt.%. All data were collected from samples in the wet state after equilibrating the disks in water for at least one day prior to testing.

**
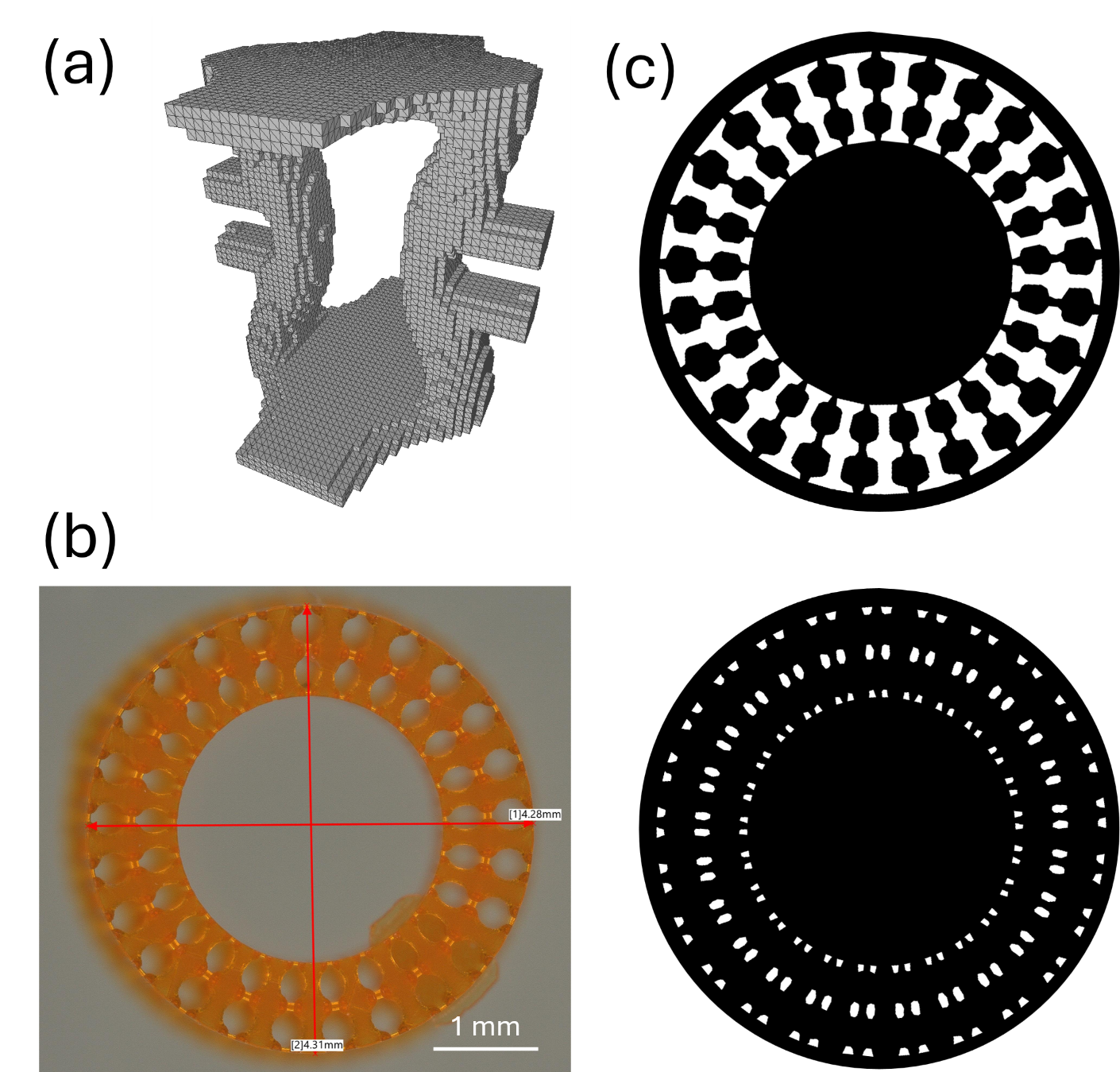
**

**Figure S3:** a) STL file of a single unit cell obtained from the design optimization described in the Methods section; b) top-down optical image of a tubular CBDA/HDDA negative Poisson’s ratio test structure. Optical inspection shows a ≈14% shrinkage in diameter, from the nominal 5 mm to ≈4.3 mm; c) corresponding x–y slices of the print design in the 001 (top) and 002 (bottom) unit cell planes.


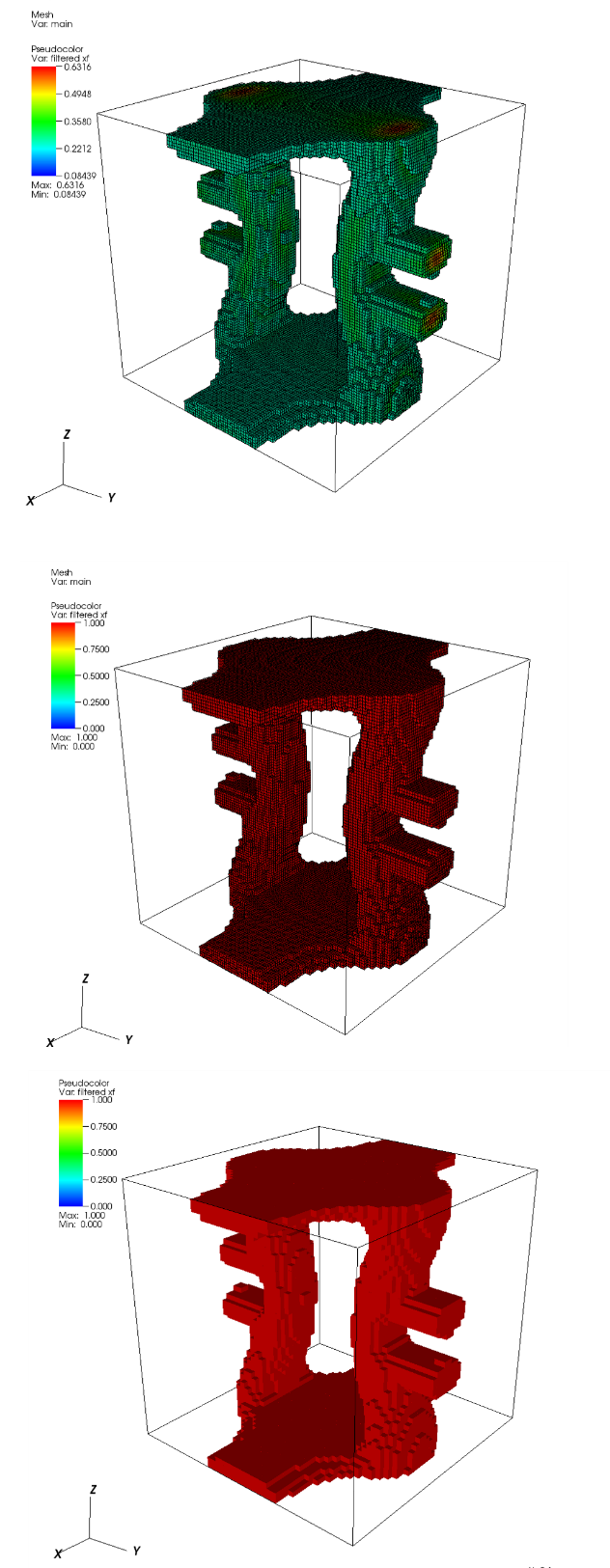


**Figure S4**. Post-processing the optimized unit cell design used in the numerical simulations. From top to bottom: (top) filtered continuous density field defining the material distribution; (middle) thresholded (binarized) version of the density field, and (bottom) the same thresholded structure visualized without mesh edges. In each panel, color indicates the density value, and low-density regions are left uncolored to allow visibility through the unit cell interior.
